# Predictors of advanced-stage presentation among patients with a diagnosis of breast and cervical cancer in Ethiopia

**DOI:** 10.1093/oncolo/oyaf019

**Published:** 2025-03-04

**Authors:** Birtukan Shewarega, Sefonias Getachew, Nigussie Assefa Kassaw, Abdu Adem Yesufe, Josephin Trabizsch, Yonas Dandena, Biruck Gashawbeza Batu, Adamu Addissie, Eva Johanna Kantelhardt, Muluken Gizaw

**Affiliations:** Department of Public Health Emergency Management, Woreda 12 Health Center, Yeka, Addis Ababa, Ethiopia; Department of Epidemiology and Biostatistics, School of Public Health, Addis Ababa University, Addis Ababa, Ethiopia; Global Health Working Group, Martin-Luther-University Halle-Wittenberg, 06112 Halle (Saale), Germany; Department of Epidemiology and Biostatistics, School of Public Health, Addis Ababa University, Addis Ababa, Ethiopia; Global Health Working Group, Martin-Luther-University Halle-Wittenberg, 06112 Halle (Saale), Germany; NCD Working Group, School of Public Health, Addis Ababa University, Addis Ababa, Ethiopia; Department of Epidemiology and Biostatistics, School of Public Health, Addis Ababa University, Addis Ababa, Ethiopia; School of Medicine, St. Paul’s Hospital Millennium Medical College, Addis Ababa, Ethiopia; Global Health Working Group, Martin-Luther-University Halle-Wittenberg, 06112 Halle (Saale), Germany; Department of Gynaecology, Martin-Luther-University, Halle-Wittenberg, 06112 Halle (Saale), Germany; School of Medicine, St. Paul’s Hospital Millennium Medical College, Addis Ababa, Ethiopia; School of Medicine, St. Paul’s Hospital Millennium Medical College, Addis Ababa, Ethiopia; Department of Epidemiology and Biostatistics, School of Public Health, Addis Ababa University, Addis Ababa, Ethiopia; Global Health Working Group, Martin-Luther-University Halle-Wittenberg, 06112 Halle (Saale), Germany; NCD Working Group, School of Public Health, Addis Ababa University, Addis Ababa, Ethiopia; Global Health Working Group, Martin-Luther-University Halle-Wittenberg, 06112 Halle (Saale), Germany; Department of Gynaecology, Martin-Luther-University, Halle-Wittenberg, 06112 Halle (Saale), Germany; Department of Epidemiology and Biostatistics, School of Public Health, Addis Ababa University, Addis Ababa, Ethiopia; Global Health Working Group, Martin-Luther-University Halle-Wittenberg, 06112 Halle (Saale), Germany; NCD Working Group, School of Public Health, Addis Ababa University, Addis Ababa, Ethiopia

**Keywords:** breast cancer, cervical cancer, advanced-stage presentation, predictors, referral pathways, financial difficulties

## Abstract

**Background:**

Breast and cervical cancers are the most common causes of cancer incidence and mortality in women in Africa. Women with breast and cervical cancers in Sub-Saharan Africa are frequently diagnosed with their disease at advanced stages. Delays in seeking health, diagnosis, and treatment are contributing factors to high mortality in Ethiopia. This study aimed to assess predictors of advanced stage presentation among patients with breast and cervical cancer attending public hospitals in Addis Ababa, Ethiopia.

**Methods:**

A cross-sectional study was conducted with a total of 418 patients at Tikur Anbessa specialized hospital and Saint Pauls’ Hospital Millennium Medical College from October to November 2021. Stages III and IV were considered advanced stages. Data were collected by reviewing medical records and face-to-face interviews with a structured questionnaire. Bivariate and multivariable analyses were performed to examine the association between independent and outcome variables.

**Results:**

A total of 269 patients with breast cancer and 149 patients with cervical cancer were included in the study, and the mean age was 44 years (SD = 10.9 years) and 50 years (SD = 11.2) years, respectively. About 66.9% of breast cancers and 71.1% of cervical cancers were diagnosed at an advanced disease stage. Rural residence (adjusted odds ratio [AOR] = 2.041, 95% CI, 1.108-3.758), indirect referral (AOR = 3.8, 95% CI, 1.485-9.946), financial difficulty (AOR = 10, 95% CI,1.859-56.495), and cancer screening recommended during their visit (AOR = 4.029 95% CI, 1.658-10.102) were independent predictors of advanced-stage presentation.

**Conclusions:**

This study revealed a high prevalence of advanced-stage breast and cervical cancer diagnosis in Ethiopia, like data collected 10 years ago, despite the introduction of a cancer control plan in 2015. For better implementation, interventions should aim to improve referral pathways, adapt screening and early detection services, and increase cancer awareness at the community level in a culturally accepted way.

**Implications for Practice**

Easing the complicated referral path for diagnostic and treatment services by arranging clear guidelines and communication mechanisms between them can help ensure timely diagnosis and appropriate management. In rural regions, efforts should be made to improve access to cancer screening, diagnosis, and treatment facilities. Implementing health insurance programs targeting patients with cancer by subsidizing diagnostic tests, and treatment costs, and providing support for transportation and accommodation expenses may have an impact on reducing the burden of advanced-stage presentation and related deaths.

## Background

Breast and cervical cancers are the most diagnosed cancers in women worldwide.^1^ Women from Sub-Saharan Africa (SSA) are disproportionally affected by both cancers, which contribute to the high burden of mortality in this region.^2^ Both cancers are responsible for 50% of the cancer burden in women in Ethiopia.^2^ Women with breast and cervical cancers in SSA are frequently diagnosed at an advanced stage.^3-5^

The 5-year survival of cervical cancer in SSA is less than 50%, and an advanced stage at diagnosis and late initiation of treatment are the 2 outstanding contributing factors to poor survival in this region.^6^ Patients with breast cancer also present with their disease at an advanced stage due to the longer interval before presentation to the first health care provider and lack of access to health facilities with diagnostic capacity in the patient’s vicinity.^7^ Moreover, complicated referrals and longer diagnostic intervals have contributed to the delay in diagnosis and initiation of curative treatment.^4^

Delays in referring these patients to the higher level are one of the contributing factors to late diagnosis and poor survival in SSA, including Ethiopia.^8^ Lack of health infrastructure, a large patient flow in the hospital, long diagnostic intervals, and patients’ repeated visits to different health facilities for diagnosis and treatment have been identified as contributing factors to the diagnosis of disease at late-stages and poor survival in SSA.^9-13^

However, despite a robust search of previous studies, we could not determine how delayed referral of those patients to secondary care is associated with presentations with advanced stage disease among patients with either breast or cervical cancer in 2 public oncology centers in Addis Ababa, Ethiopia. Therefore, the present study explains the association between the direct referral path and advanced-stage presentation. We also sought to identify common individuals, health systems, and clinical factors contributing to diagnosis in late stages among patients with breast and cervical cancer diagnosed at a late stage.

## Methods

### Study design and setting

An institutional based, cross-sectional study was conducted in Tikur Anbessa specialized hospital (TASH) and Saint Pauls’ Hospital Millennium Medical College (SPHMMC), 2 public referral hospitals (TASH and SPHMMC) in Addis Ababa. Tikur Anbessa Specialized Hospital is the largest cancer treatment center including radiation treatment. Saint Paul’s Hospital Millennium Medical College is the second referral and teaching hospital offering cancer treatment following TASH.

### Study participants

A total of 418 patients with histopathologically confirmed breast and cervical cancer (aged > 18 years) who were diagnosed at least 1 month prior to the data collection period and on follow-up for treatment were enrolled in the study. Patients with breast and cervical cancer who were critically ill or missing dates regarding pathological diagnosis, cancer staging, and symptom onset were excluded from the study.

## Sampling technique and sample size

The hospitals were selected purposively by the criteria of having oncology services. Consecutive sampling was used to enroll study subjects. The sample size was calculated using the single population proportion formula with Epi info version 4.1 software using the parameters 95% CI, 80% power, 5% margin of error, and proportion advanced-stage presentation of cervical cancer patient study conducted at TASH p = 55.2%.^14^

## Variables and measurements

The following Aarhus statement criteria were used for classifying patient and diagnostic intervals:

The **patient interval/appraisal interval** was defined as the time interval from first symptom recognition (the time point when first bodily changes and/or symptoms were noticed) to presentation to the first health care provider, and a **patient appraisal/patient delay** was defined as a patient interval greater than 3 months (>90 days).^15^

The **diagnostic interval** was defined as the interval from the date of first clinical presentation to the date of pathological diagnosis. The date of diagnosis was taken from the patient’s pathology report. A **diagnostic delay** was defined as a time interval between the first clinical presentation and the date of pathological diagnosis (diagnostic interval) greater than 30 days.^4^

**Direct referral** occurred when patients with a given set of symptoms were referred within 2 weeks and obtained a diagnosis of cancer within 2 weeks in the first receiving facility.^16^

**Financial difficulty** was defined as financial hardship incurred for transportation and medical expenses.

## Data collection and data quality assurance

Data were collected by a review of medical records and face-to-face interviews using structured questionnaires. Nurses who were working in the oncology department collected data, and training was offered for 3 days. Pretesting was performed prior to data collection in 20 respondents.

## Data analysis

Descriptive statistics were used to measure the frequency and proportion of advanced disease presentation. Bivariate and multivariable logistic regression analyses were used to identify predictors of advanced stage diagnosis.

## Ethical consideration

Ethical clearance was secured from the Institutional Review Board of the School of Public Health, College of Health Science, and Addis Ababa University (SPH/1119/2021). Verbal consent was obtained from all study participants. The confidentiality of participants was kept by not sharing their personal identifiers in all research processes.

## Result

### Socio-demographic characteristics of the respondents

The mean age was 44 years (SD = 10.9 years) and 50 years (SD = 11.2) for patients with a diagnosis of breast cancer and cervical cancer, respectively. Around 234 (56%) of the study participants were from urban areas, while 44% were rural dwellers. A quarter (106; 25.4%) of participants could not read or write, and 185 (44.2%) had attended secondary school or above. Around 267 (63.9%) respondents were married and 310 (74.2%) were Orthodox Christian by religion (Table 1).

**Table 1.** Socio-demographic characteristics of respondents in TASH and SPHMMC, 2021.

Variable	Category	Frequency (*n* = 418)	Percent (%)
Age	≤50	272	65.1
	≥51	146	34.9
Religion	Orthodox	310	74.2
	Muslim	63	15.1
	protestant	45	10.8
Residence	Urban	234	56.0
	Rural	184	44.0
Educational	Cannot read and write	106	25.4
	Can read and write	38	9.1
	Elementary	89	21.3
	Secondary and above	185	44.2
Marital status	Married	267	63.9
	Single	19	4.5
	Divorced	70	16.7
	widowed	62	14.8
Occupation	Housewife	217	51.9
	Government employee	80	19.1
	Private employee	39	9.3
	Merchant	13	3.1
	Daily laborer	12	2.9
	Student	2	.5
	Retired	9	2.2
	Farmer	46	11.0

Close to half (132; 49.07%) of patients with breast cancer ≤50 years of age had advanced-stage presentations, whereas only 48 (17.84%) respondents in the >50 age group had advanced-stage presentations. However, there was an equal proportion of patients with cervical cancer with advanced-stage presentations, 53 (35.57%), in both age groups. Around 36.43% of patients with breast cancer and 34.23% of patients with cervical cancer had a monthly income of less than 5000 Ethiopian Birr (ETB) (Table 2).

**Table 2.** Socio-demographic characteristics of participants according to disease presentation at diagnosis in TASH and SPHMMC, 2021.

Variable	Category	Early breast cancer (frequency [%])	Late breast cancer (frequency [%])	Early cervical cancer (frequency [%])	Late cervical cancer late (frequency [%])
Age	≤50	62 (23.04%)	132 (49.07%)	25 (16.78%)	53 (35.57%)
	>50	27 (10.03%)	48 (17.84%)	18 (12.08%)	53 (35.57%)
Religion	Orthodox	62 (23.04%)	134 (49.82%)	31 (20.51%)	83 (55.70%)
	Muslim	18 (6.69%)	25 (9.29%)	8 (5.37%)	12 (8.05%)
	protestant	9 (3.35%)	21 (7.81%)	4 (2.68%)	11 (7.38)
Residence	Urban	65 (24.16%)	101 (37.55%)	29 (19.46%)	39 (26.17%)
	Rural	24 (8.92%)	79 (29.37%)	14 (9.40%)	67 (44.97%)
Educational status	Cannot read and write	44 (16.36%)	94 (34.94%)	12 (8.05%)	47 (31.54%)
	Can read and write	21 (7.81%)	44 (16.36%)	5 (3.36%)	14 (9.40%)
	Elementary	10 (3.72%)	17 (6.32%)	8 (5.37%)	21 (14.09%)
	Secondary and above	14 (5.2%)	25 (9.29%)	18 (12.08%)	24 (16.11%)
Marital status	Married	55 (20.45%)	110 (40.89%)	31 (20.81%)	71 (47.65%)
	Single	4 (1.49%)	11 (4.09%)	0 (0%)	4 (2.38%)
	Divorced	16 (5.95%)	32 (11.89%)	6 (4.02%)	16 (10.74%)
	Widowed	14 (5.2%)	27(10.04%)	6 (4.02%)	15 (10.07%)
Occupation	Housewife	44 (16.36%)	94 (34.94)	23 (15.44%)	56 (37.58%)
	Government employee	21 (7.81%)	44 (16.36%)	7 (4.69%)	8 (5.37%)
	Private employee	10 (3.72%)	17 (6.32%)	3 (2.02%)	9 (6.04%)
	Merchant	2 (0.7%)	8 (2.974%)	0 (0%)	3 (2.02%)
	Daily laborer	3 (1.12%)	3 (1.12%)	2 (1.34%)	4 (2.68%)
	Student	1 (0.4%)	1 (0.4%)	2 (1.34%)	2 (1.34%)
	Retired	3 (1.12%)	2 (0.7%)	6 (4.03%)	24 (16.11%)
	Farmer	5 (1.86%)	11 (4.09%)	23 (15.44%)	56 (37.58%)
Monthly income (ETB)	≤5000	53 (19.7)	98 (36.43%)	23 (15.44%)	51 (18.95%)
	>5000	36 (13.38%)	82 (30.48%)	20 (13.42%)	55 (36.91%)

### Clinical characteristics of respondents

About half (51.3%) and 71 (47.7%) patients with a diagnosis of breast and cervical cancer were on chemotherapy alone, respectively. About 38.3% of patients with cervical cancer had been treated with chemotherapy and radiation therapy. Most patient with a diagnosis of breast cancer (66.9%) were diagnosed with their disease at an advanced stage (III and IV). Similarly, 71.1% of patients with a diagnosis of cervical cancer were presented with disease in advanced stages. Screening awareness was present in 108 (40.1%) and 43 (29.8%) patients with breast and cervical cancer, respectively. Among them, only 0.4% of patients with a diagnosis of breast and 6% of patients with a diagnosis of cervical cancer had a history of screening before diagnosis. Almost 85% of patients with breast cancer had complained of a breast lump at presentation. Similarly, 92 (62.2%) patients with cervical cancer had a presenting complaint of bloody vaginal discharge followed by pelvic pain (22.3%) or nonbloody vaginal discharge (10.8%).

### Predictors of advanced-stage presentation among breast and cervical cancer patients

Patients with breast and cervical cancer who had been referred indirectly were more likely to present with their disease at an advanced stage than those who had been referred directly (AOR of 3.843; 95% CI, 1.485-9.946). Similarly, rural residents were 2 times more likely to be diagnosed at an advanced disease stages than those who lived in urban areas (AOR of 2.041; 95% CI, 1.108-3.758). Patients with either breast or cervical cancer who had a patient delay >3 months were 2.5 times more likely to have advanced-stage presentations than their counterparts (AOR = 2.479; 95% CI, 1.078-5.704). Moreover, patients with breast and cervical cancer who reported financial difficulties were more likely to have advanced-stage presentations than their counterparts (AOR = 10.102; 95% CI, 1.658-10.248). A substantial percentage, 73.7% (308) of patients with a diagnosis of breast and cervical cancer were unaware of the symptoms before being diagnosed, causing the patient to appear late for diagnosis and treatment. Those women who had low awareness about screening and disease itself were ~4 times more likely to have advanced-stage presentations than who had higher awareness (AOR = 4.092; 95% CI, 1.078-5.704). Our findings show that 45.3% of participants had a delay in diagnosis following the initial medical consultation. The health system (delayed referral and waiting test results) or the health care provider (misdiagnosis) were responsible for the delay in diagnosis (Table 3).

**Table 3.** Factors associated with advanced-stage presentation among breast/cervical cancer patients in TASH and SPHMMC Addis Ababa, Ethiopia, 2021.

Variable	Stage		COR with 95% CI	AOR with 95% CI	*P*-value
	Early	Advanced			
Referral status **Direct** **Indirect**	108 (25.83%)	57 (13.63%)	1.0	1.0	
	24 (5.74%)	229 (54.78%)	18.079 (10.652-30.683)	3.843 (1.485-9.946)	.006
Lack of money **Yes** **No**	2 (0.478%)	20 (4.78%)	4.887 (1.125-21.226)	10.248 (1.859-56.495)	.008
	130 (31.1%)	266 (63.63%)	1.0	1.0	
Cancer screening recommended during their visit **Yes** **No**	119 (28.46%)	97 (23.20%)	1.0	1.0	
	13 (3.11%)	189 (45.21%)	17.836 (9.569-33.244)	4.092 (1.658-10.102)	.002
Patient delay ≥ 3 month **Yes** **No**	87 (20.81%)	221 (52.87%)	2.943 (1.697-5.101)	2.479 (1.078-5.704)	.033
	45 (10.76%)	65 (15.55%)	1.0	1.0	
Residence **Urban** **Rural**	94 (22.48%)	140 (33.49%)	1.0	1.0	
	38 (9.09%)	146 (34.92%)	2.580 (1.657-4.015)	2.041 (1.108-3.758)	.022

^*^Only variables with *P* <.25 were included in the multivariable analysis.

## Discussion

This study examined the predictors of advanced-stage presentations among patients with a diagnosis of breast and cervical cancer in Ethiopia. About two thirds of patients with breast and cervical cancer had advanced disease stage at the time of diagnosis. An indirect referral path, financial difficulty, rural residence, a low awareness level, and a patient delay >3 months were independent predictors of advanced-stage presentations.

The findings of such high proportions of advanced breast cancer disease at diagnosis were consistent with those of northeast Ethiopian (65.7%), Nigerian (67.7%), and studies from India (68%).^12,17,18^ However, they were lower than those reported in a study conducted in northwest Ethiopia (71.2%)^3^ and higher than those of studies conducted in South Africa (51%), Pakistan (59%), and Germany (51.6%).^19-21^ The proportion of patients with cervical cancer with an advanced stage of disease was nearly similar to those in studies conducted in Nigeria (72.8%)^15^ and Sudan (72%)^22^ and higher than the 56.8% of patients presenting with advanced disease in a study conducted in TASH in 2021^23^ and a population-based cross-sectional study conducted in Addis Ababa (60.4%).^24^

According to the findings, a large proportion of women with breast and cervical cancer were unaware of the symptoms prior to their diagnosis, resulting in a delay in patient presentation for diagnosis and treatment. The proportion of patients who were delayed accounts for 73.7% (308), which is higher than the 50.5% in studies conducted at Dessie Referral Hospital, northeast Ethiopia’s only oncology unit,^25^ and the 65.3% in northern India.^12^ Another study from Ethiopia at TASH in 2010 showed that almost half of the study participants (*n* = 33; 47.8%) said that women in Ethiopia typically know nothing about breast cancer and, in fact, have often never heard of the disease at all. This difference might be due to the difference in sample size of the studies, adoption of different cutoff points for classifying patient delay, and a difference in study populations.^26^

Our study showed the effects of a patient delay of >3 months on finding advanced-stage disease at diagnosis in patients with breast and cervical cancer. The longer the patient delay time, the more likely the patient was diagnosed at an advanced disease stage. Patients who had a presentation delay of >3 months were 2.4 times more likely to be diagnosed at an advanced stage than those who had no patient delay. Disease progression is expected during a delay. A similar finding was reported in studies conducted in northeast Ethiopia, in which 50.5% (103) women with breast cancer were delayed for >3 months. Out of these delayed women, 75 (72.8%) were diagnosed at a late stage (*P* <.030).^25^ Similarly, a longer patient interval in patients with cervical cancer is associated with more advanced FIGO stages at diagnosis, according to a study performed at TASH.^14^

According to our findings, the delay in diagnosis after the initial medical consultation was attributed to either the health system (delayed referral and waiting test result) or the health care provider (misdiagnosis), in 45.3% of cases. A similar result was reported from Egypt (37%).^27^

The study revealed that the odds of presenting with advanced-stage disease were 2.0 times greater in patients residing in rural than in urban areas. This finding is nearly identical to that of a 2021 study in TASH^23^ and like those of studies conducted in Sudan,^22^ Nigeria,^17^ and northwest Ethiopia.^3^ This is probably because women who live in places with limited access to health care or who travel longer distances for treatment are more likely experience a delay. Additionally, cancer literacy is probably lower in rural settings. However, this finding contrasts with those of a study on patients with a diagnosis of cervical cancer conducted in 2019 at TASH Ethiopia,^14^ which found that the place of residency was not a predictor of advanced-stage disease presentation. This discrepancy could be explained by differences in study methods, settings, sample sizes, and research participants.

Our study found no evidence of associations between level of education, age, or marital status and advanced-stage presentation. This finding is supported by previous studies in Ethiopia and Nigeria, where age was not associated with an advanced disease stage.^14,27^ Nonetheless, this result is in contrast with other studies in Nigeria and Ethiopia, which reported that age and level of education were strong determinants of stage at diagnosis in cervical cancer.^23,27^ Women without a high school diploma and those who were above the age of 60 had a considerably higher risk of presenting with advanced-stage disease than their counterparts.^23,27,28^ This disparity could be related to differences in the study location and participants.

Consistent with findings from prior studies,^16,19^ indirect referral significantly affected advanced-stage presentation in the current study. The odds of advanced-stage presentation were higher among women with breast and cervical cancer who were referred indirectly (AOR 3.843, 95% CI, 1.485-9.946); this probably reflects the longer duration of referral.

Our study has some limitations. First, even though this study involved a relatively large sample size, due to the nature of a cross-sectional study, the establishment of a causal relationship was not possible. Second, the retrospective nature of information collection from patients on the dates of symptom recognition and medical consultations is prone to recall bias and could have affected the measurement of intervals and other variables, but we have tried to minimize it by using standard tools, carefully designing the questionnaire and training the data collectors.

## Conclusion

Despite the efforts made by the government to improve cancer care and patient outcomes, the majority of patients with a diagnosis of breast and cervical cancer present with their disease at an advanced stage. Shortening the referral pathways and designing a direct “fast track” referral for diagnosis and treatment could help to improve the delay in diagnosis and treatment initiation. Implementing diagnostic walk-in centers could be an option to achieve this. Awareness-raising activities should target the general population to promote screening for both cancers and facilitate early detection. Special attention must be given to vulnerable segments of the population, such as rural residents and women with low incomes.

## Data Availability

The data will be available upon the reasonable requests to the corresponding author.
